# Indeterminate Lesions of the Testis in Klinefelter Syndrome—Should We Be Concerned?

**DOI:** 10.3389/frph.2021.629418

**Published:** 2021-06-24

**Authors:** Aneirin Rhys Potter, Davide Prezzi

**Affiliations:** ^1^School of Medicine, King's College London, London, United Kingdom; ^2^School of Biomedical Engineering and Imaging Sciences, King's College London, London, United Kingdom; ^3^Department of Radiology, Guy's and St Thomas' NHS Foundation Trust, London, United Kingdom

**Keywords:** Klinefelter, infertility—male, testis (MeSH), testis neoplasm, contrast—enhanced ultrasonography, ultrasound elasticity imaging, testicular lesion

## Introduction

The diagnostic workup of male infertility commonly includes scrotal ultrasound as an adjunct to physical examination. Testicular volume and morphology are readily evaluated; signs of testicular dysgeneses, such as heterogeneous echotexture and microcalcifications, and non-palpable focal testicular lesions can be identified. Additional sonographic findings include signs of spermatic obstruction, such as dilatation of the rete testis, enlarged epididymis, and absent vas deferens. In men with varicocele, the presence of venous reflux during Valsalva maneuver is assessed using Doppler (1).

The widespread use of ultrasound, combined with improved resolution of modern transducers, has led to increased detection of focal non-palpable testicular lesions. Their prevalence is higher by 20% in men with azoospermia compared with the general population (1, 2). Despite this, recent large cohort studies show that patients affected by Klinefelter syndrome (KS) are at no higher risk of testicular germ cell tumors than the general population (3, 4).

**Figure 1:**
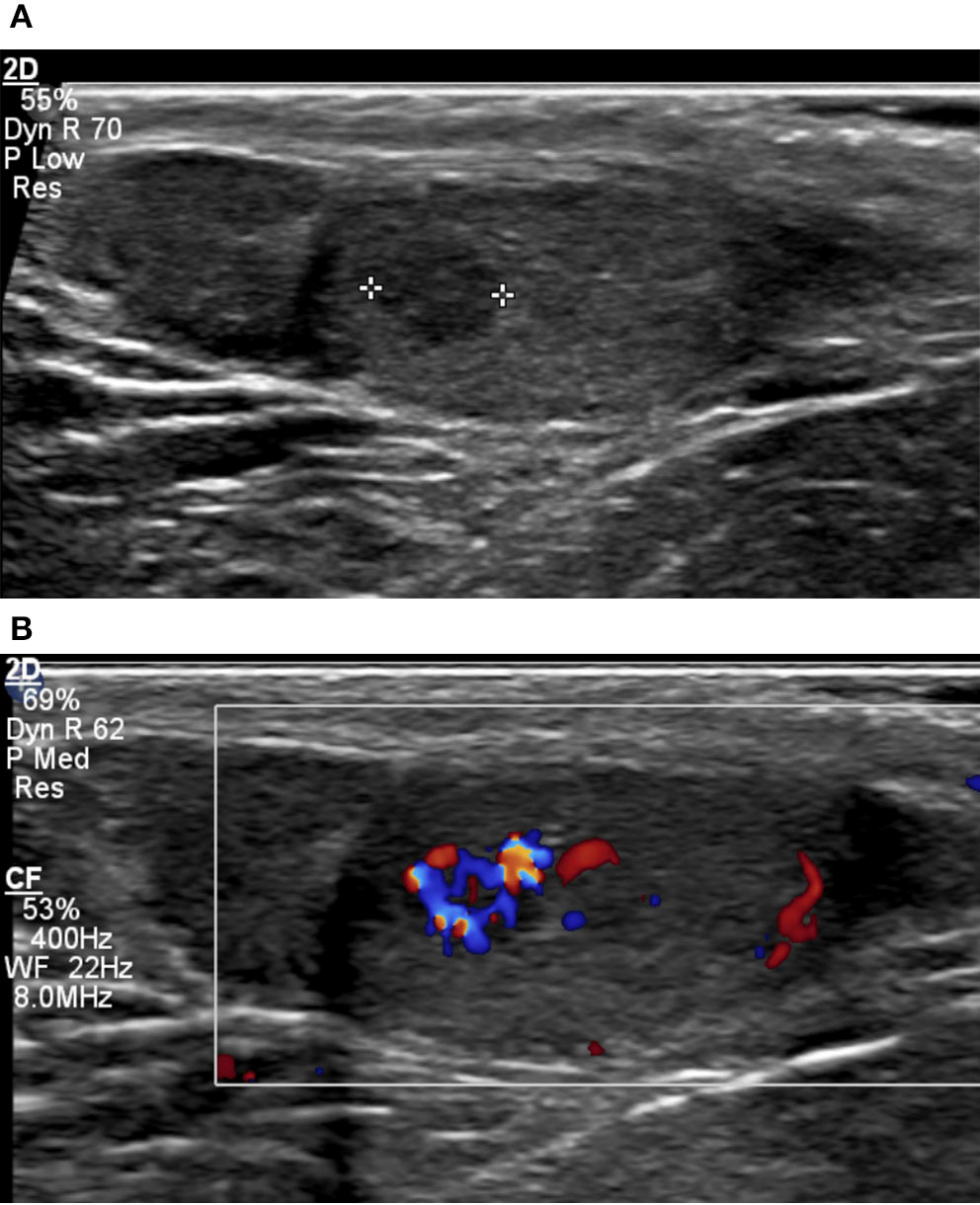
Small-volume testis (2 ml) in a 36-year-old patient with KS, characterized by coarse background echotexture **(A)**. A 4-mm hypoechoic lesion with markedly increased vascularity on color Doppler **(B)** was found to correspond to nodular Leydig cell hyperplasia on surgical biopsy.

Typical sonographic features in KS men include small-volume testes with a coarse or nodular echotexture (Figure 1). In a single-center retrospective study, measurable nodules were observed in 42 out of 67 KS men. Five men presenting with one or more nodules measuring more than 3 mm in diameter were referred for surgery: All nodules were found to be benign Leydig cell tumors (LCT) at histopathology. About 18 men undergoing testicular biopsy combined with extraction of spermatozoa showed dominant Leydig cell hyperplasia, Sertoli cell involution, and seminiferous tubule degeneration (5).

Leydig and Sertoli cell tumors are the main solid lesions to be differentiated from germ cell tumors, both belonging to the family of sex cord-stromal tumors of the testis. LCT is the most frequent sex cord tumor, accounting for up to 3% of testicular tumors, and is benign in over 90% of cases. Small lesions typically have a slow, indolent course. Leydig cell hyperplasia is a benign condition that overlaps with LCT; it is commonly detected in cryptorchid testes and in KS. Unlike in LCT, Leydig cells are arranged in a more diffuse micronodular pattern within the testicular interstitium. Well-preserved or atrophic tubules can be observed among the hyperplastic nodules, a feature that is absent in LCT (6).

Leydig cell tumors are typically round, sharply defined hypoechoic lesions with homogeneous echotexture. They cannot be reliably differentiated from germ cell tumors purely based on morphological sonographic features. They have a characteristic Doppler appearance, consisting of moderate hypervascularity relative to the adjacent parenchyma (7).

The combined use of morphological ultrasound, color Doppler, contrast-enhanced ultrasound (CEUS), and real-time elastography, referred to as multiparametric ultrasound, has been explored in recent years to characterize non-palpable focal testicular lesions with promising results. Increased stiffness has been found on strain elastography in both malignant and benign neoplastic lesions, including LCT, whereas non-neoplastic lesions tend to be soft (8). On CEUS, neoplastic lesions show a more rapid wash-in and wash-out compared to non-neoplastic lesions and normal testicular parenchyma. Rapid contrast wash-out was a distinctive feature of malignant lesions in a prospective study by Isidori et al. (7), whereas delayed or synchronous wash-out relative to parenchyma was typical of benign lesions including LCT (7).

Optimum management of non-palpable focal testicular lesions is a matter of debate in the urological community. A lack of clinical and imaging parameters to distinguish benign from malignant lesions has led to overtreatment by means of radical orchidectomy. A systematic review by Giannarini et al. (9) reported on the safety of testis sparing surgery for all proven benign testicular neoplasms and for selected malignant tumors in solitary testes in combination with adjuvant radiotherapy. Increased use of ultrasound follow-up is also being adopted as an alternative to surgery (10). In a large single-center cohort of 120 infertile men with non-palpable testicular lesions <10 mm, Bieniek et al. (11) found most lesions to remain stable in size over a mean follow-up of 1.3 years; only 6 out of 18 men undergoing surgery for progressive lesions had malignant tumors at histopathology.

## Conclusion

Indeterminate lesions of the testis are common sonographic findings in infertile men, including those affected by KS. Published data reflecting the experience of a few specialized centers show that most non-palpable lesions correspond to benign LCT; these lesions can be safely followed up or biopsied at the time of microsurgical sperm retrieval. Multiparametric ultrasound shows potential for improved lesion characterization and may assist in their management in future. Unnecessary surgery or prolonged imaging follow-up could be avoided in the presence of lesions with sonographic features favoring benignity; prompt organ sparing surgery could be directed to lesions with malignant features.

## Author Contributions

AP drafted the manuscript. DP edited the manuscript to its submitted form. All authors contributed to the article and approved the submitted version.

## Conflict of Interest

The authors declare that the research was conducted in the absence of any commercial or financial relationships that could be construed as a potential conflict of interest.
